# Variations in Pulse-Oxygen Saturation and Physical Development within the First 2 Hours after Birth Among Healthy Term Neonates Different Altitudes

**DOI:** 10.33549/physiolres.935666

**Published:** 2026-04-01

**Authors:** Kun DU, Yong-Li YANG, Guo-Yun LI, Hui MAO, Ming-Cai QIN, Yin-Zhen LAI, Cai-Ren ZHUO-MA, Yang-Fang LI, Xiao-Mei LIU

**Affiliations:** 1 Department of Neonatology, Kunming Children’s Hospital, Kunming, Yunnan Province, China; 2 Department of Pediatrics, Lijiang Maternal and Child Health Hospital, Lijiang, Yunnan Province, China; 3 Department of Pediatrics, Xishuangbanna State Hospital, Xishuangbanna, Yunnan Province, China; 4 Department of Obstetrics, Affiliated Hospital of Yunnan University, Kunming, Yunnan Province, China; 5 Department of Pediatrics, Dechen Tibetan Autonomous Prefecture People’s Hospital, Shangri-La, Yunnan Province, China; 6 Department of Pediatrics, Shangri-La County Maternal and Child Health Hospital, Shangri-La, Yunnan Province, China; 7 Department of Pediatrics, Yushu State People’s Hospital, Yushu, Yunnan Province, China

**Keywords:** Different altitudes, Healthy newborns, Full-term infants, Percutaneous oxygen saturation, Physical growth, Pulse oximetry monitoring

## Abstract

This study aimed to examine early postnatal changes in pulse oxygen saturation (SpO_2_) and physical growth parameters during the first 2 hours following birth among healthy term neonates born at varying altitudes. A total of 963 healthy term neonates delivered in five regions at varying altitudes were enrolled. SpO_2_ levels were measured at multiple time points within the initial 2-hour postnatal period. Physical measurements including birth weight, body length, and weight-to-length ratio, were assessed and compared across altitudinal groups. Significant differences in SpO_2_ values were identified among neonates born at different altitudes (*p* < 0.05). SpO_2_ demonstrated a time-dependent increase following birth, with a general decrease in saturation levels observed at higher altitudes. No statistically significant differences were found in birth weight or weight-to-length ratio between groups. However, body length differed significantly across regions (*p* < 0.001). Healthy term neonates experience progressive increases in SpO_2_ during the early postnatal period, irrespective of altitude. Neonates residing at high altitudes, including those of Tibetan ethnicity, exhibit favorable physical development indicators.

## Introduction

Pulse oximetry constitutes an essential noninvasive method for assessing the requirement for oxygen therapy in neonates and serves as an effective modality for the early detection of congenital heart defects [1]. As arterial oxygen content is modulated by altitude, reference ranges for peripheral oxygen saturation (SpO_2_) should be adapted according to altitude-specific physiological conditions. Accordingly, altitude must be carefully considered when determining whether pulse oximetry readings in newborn populations are within normal limits.

Among neonates born at sea level with normal respiratory function, SpO_2_ increases rapidly after birth, rising from approximately 50 % at delivery to over 90 % within the first 10 minutes, and exceeding 95 % by 15 minutes of life [2]. This increase is attributed to several physiological changes occurring postnatally, including clearance of pulmonary fluid, reduction in pulmonary vascular resistance, and constriction and functional closure of the ductus arteriosus, mediated by increased arterial oxygen partial pressure [3]. Notably, arterial oxygen partial pressure decreases with increasing altitude due to reduced atmospheric pressure, thereby diminishing the diffusion gradient for oxygen from alveolar air into the bloodstream and resulting in hypobaric hypoxia.

Consequently, neonates born in high-altitude regions may exhibit lower SpO_2_ values during the immediate postnatal period compared with neonates born at lower altitudes. Moreover, chronic exposure to a relatively hypoxic intrauterine environment during high-altitude pregnancies may affect somatic growth parameters at birth, contributing to regional variability in physical development. Therefore, the application of altitude-specific reference standards is necessary for accurately assessing neonatal oxygenation status and establishing appropriate intervention thresholds.

Despite these physiological distinctions, standardized criteria are frequently applied across altitude levels in clinical settings, and there remains a lack of consensus regarding the normative SpO_2_ range for healthy neonates born at high altitudes within the first few hours of life [4].

The present study aimed to evaluate SpO_2_ during the first 2 hours after birth and assess physical development at birth among neonates from regions at different altitudes. Specific objectives included the establishment of altitude-specific SpO_2_ reference values, characterization of temporal trends in postnatal oxygen saturation, and determination of an optimal SpO_2_ cutoff value for neonates in high-altitude settings between 30 and 120 minutes of life. These findings may inform the development of more appropriate clinical assessment and intervention strategies tailored to this population.

## Methods and Study Participants

This multi-center study was conducted across five affiliated hospitals participating in the Highland Neonatal Medical Alliance. Participants in this study were drawn from five hospitals affiliated with the Plateau Neonatal Medicine Alliance, situated at varying altitudes: Xishuangbanna (552 m), Kunming (1890 m), Lijiang (2416 m), Diqing (3280 m), and Yushu (4493 m).

Eligible participants comprised live-born, healthy, full-term neonates delivered in the obstetrics departments of the aforementioned hospitals. Inclusion criteria required singleton neonates born between 37 and 42 weeks of gestation who demonstrated stable vital signs. Exclusion criteria were: (1) the presence of visible congenital anomalies; (2) respiratory distress necessitating oxygen therapy; and (3) evidence of intrauterine distress or a 1-minute Apgar score ≤7 requiring resuscitative interventions.

This study was approved by the Ethics Committee of Kunming Children’s Hospital, the lead institution of the Highland Neonatal Medical Alliance (approval number: 2022-03-275-K01). Written informed consent was obtained from the legal guardians of all enrolled neonates after being provided with comprehensive information regarding the purpose and significance of SpO_2_ monitoring. The study is registered at ClinicalTrials.gov (registration number: NCT05468515).

Postnatal measurements of SpO_2_, birth weight, and body length were obtained at three time intervals: 0–10 minutes, 10–30 minutes, and 60–120 minutes after birth.

### SpO_2_ monitoring

SpO_2_ was measured using a neonatal pulse oximeter by trained personnel in a quiet environment while the neonate remained calm. A neonatal-specific pulse oximetry probe was placed on the right wrist. Readings were recorded after the device displayed a stable waveform and numeric value for a duration of 10–15 seconds.

### Assessment of physical parameters

#### Birth Weight

Birth weight was measured within 2 hours postpartum using a calibrated electronic infant scale (maximum capacity: 20 kg; accuracy: 10 g).

#### Body Length

Body length was measured with a flexible measuring tape (range: 100 cm; precision: 0.1 cm) extending from the crown of the head to the heel, with the neonate placed in a supine position.

A dual-observer, double-measurement protocol was implemented. If the second measurement differed from the first by less than 10 g (weight) or 0.5 cm (length), both values were retained. In cases where the discrepancy exceeded these thresholds, a third measurement was taken, and the two closest values were selected for analysis. The weight-to-length ratio (kg/m) was calculated accordingly.

### Statistical methods

Statistical analyses were conducted using R software (version 4.4.1; R Foundation for Statistical Computing, Vienna, Austria). A two-sided *p*-value < 0.05 was considered statistically significant. When multiple comparisons were performed, the Bonferroni correction method was applied to adjust the significance threshold.

Continuous variables with a normal distribution were expressed as mean ± standard deviation (χ̄ ± SD), whereas non-normally distributed variables were presented as median and interquartile range [M (IQR)]. Categorical variables were summarized using frequency and percentage [n (%)]. Differences in sex and ethnicity across regions were analyzed using the chi-squared (χ^2^) test. Non-parametric tests were employed to evaluate inter-regional differences in birth weight, body length, and weight-to-length ratio. SpO_2_ distributions across regions were compared using the Kruskal-Wallis test; with further multiple comparisons performed using the Nemenyi test.

## Results

### General characteristics

A total of 963 newborns were included in this study: 215 from Xishuangbanna, 131 from Kunming, 382 from Lijiang, 107 from Diqing, and 128 from Yushu. Among them, 495 were male (51.19 %) and 470 were female (48.81 %), yielding a male-to-female ratio of 1.05:1. There were 348 Han infants (36.14 %), mainly from Kunming, Xishuangbanna, and Lijiang, and 615 ethnic minority infants (63.86 %), including 200 Dai (20.76 %), 112 Naxi (11.64 %), and 303 Tibetan (31.46 %) infants. The Dai and Naxi groups were primarily distributed in Xishuangbanna and Lijiang, whereas the Tibetan group was mainly distributed in the high-altitude regions of Diqing and Yushu. No statistically significant differences were found in sex distribution among regions; however, the ethnic distribution differed significantly (χ^2^ = 210.67, *p* < 0.001) (Table 1).

### Comparison of SpO_2_ at Different Altitudes

#### Postnatal changes in SpO_2_ among neonates at different altitudes

**Fig. 1:**
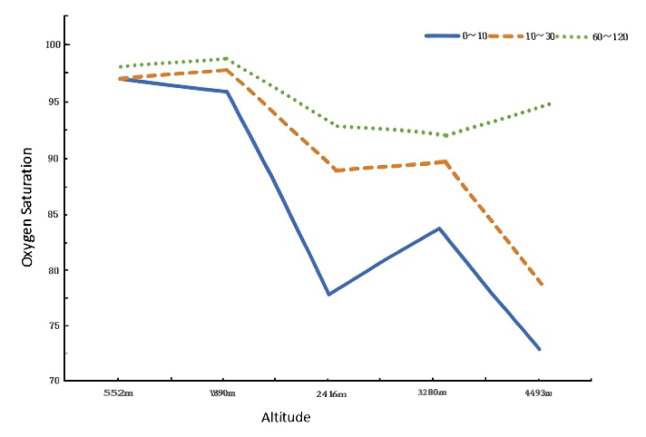
Changes in SpO_2_ at different altitudes. Xishuangbanna, 552 meters; Kunming, 1890 meters; Lijiang, 2416 meters; Diqing, 3280 meters; Yushu, 4493 meters. *p* < 0.05 indicates significant differences.

**Fig. 2:**
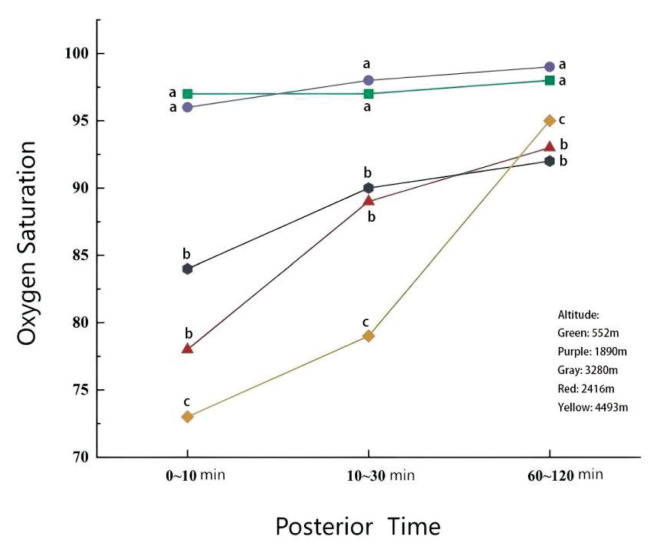
Changes in SpO_2_ over time. Xishuangbanna, 552 meters; Kunming, 1890 meters; Lijiang, 2416 meters; Diqing, 3280 meters; Yushu, 4493 meters. *p* < 0.05 indicates significant differences. Identical letters indicate no statistical difference between the two at this time point; different letters indicate a statistical difference between the two during this time period.

SpO_2_ values indicated a significant inverse association with altitude, with lower readings observed at higher elevations (Fig. 1). A general ascending trend in SpO_2_ was observed over time across all regions (Fig. 2). However, deviations from altitude-correlated trends were noted at certain time points. For example, neonates in Diqing (3280 meters) exhibited higher SpO_2_ values than those in Lijiang (2416 meters) during the 0–10 minute and 10–30 minute periods. Additionally, neonates in Kunming (1890 meters) demonstrated higher SpO_2_ values than those in Xishuangbanna (552 meters) at 10–30 minutes and 60–120 minutes postnatally. Notably, neonates in Yushu (4493 meters) exhibited the lowest SpO_2_ readings at 0–10 minutes but demonstrated substantial increases by 60–120 minutes, surpassing the corresponding values observed in Lijiang (2416 meters) and Diqing (3280 meters).

### Inter-regional differences in SpO_2_

**Fig. 3:**
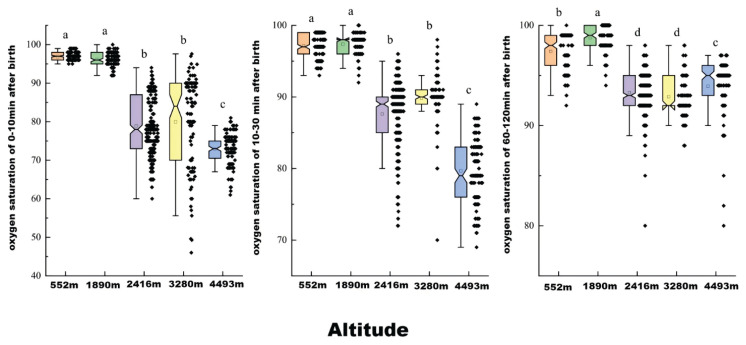
Distribution of oxygen saturation in newborns at various postnatal time points. Identical letters indicate no significant difference; different letters indicate a significant difference.

As presented in Table 2 and visualized in Figure 3, the Nemenyi test identified significant differences in SpO_2_ levels across various regional pairings. During the 0–10 and 10–30 minute intervals, no significant differences were observed between Xishuangbanna (552 meters) and Kunming (1890 meters), or between Lijiang (2416 meters) and Diqing (3280 meters). However, SpO_2_ values in both Xishuangbanna (552 meters) and Kunming were significantly higher than those measured in Lijiang, Diqing, and Yushu (4493 meters) (all *p* < 0.001).

At 60–120 minutes postnatal, no significant differences were observed between Lijiang (2416 meters) and Diqing (3280 meters). Nevertheless, multiple regional comparisons revealed significant differences, including Kunming (1890 meters) versus Xishuangbanna (552 meters), Kunming (1890 meters) versus Lijiang (2416 meters) / Diqing (3280 meters) / Yushu (4493 meters), and Xishuangbanna (552 meters) versus Lijiang (2416 meters) / Diqing (3280 meters) / Yushu (4493 meters) (all *p* < 0.001). The overall distribution trend of SpO_2_ values at 60–120 minutes was as follows: “1890 meters > 552 meters > 4493 meters > 2416 meters” and “1890 meters > 552 meters > 4493 meters > 3280 meters.”

### Inter-regional differences in birth anthropometry

No statistically significant differences in birth weight were observed among neonates from the different regions. However, body length differed significantly (*p* < 0.001), while weight-to-length ratio did not differ significantly between regions. Pairwise comparisons indicated that neonates in Xishuangbanna (552 meters) had significantly different body lengths compared to those in Kunming (1890 meters), Lijiang (2416 meters), and Yushu (4493 meters). Similarly, neonates in Lijiang (2416 meters) differed significantly in body length from those in Diqing (3280 meters) and Yushu (4493 meters) (Table 3).

## Discussion

Pulse oxygen saturation monitoring measures changes in arterial oxygen levels by detecting how skin tissue absorbs light at different wavelengths. Transcutaneous SpO_2_ is a cost-effective, convenient, and non-invasive monitoring method that allows early detection of neonatal hypoxemia. It is widely used not only for patient monitoring and guiding oxygen therapy [5] but also for early screening of congenital heart disease [6]. Continuous dynamic monitoring of SpO_2_ also provides an essential reference for adjusting the fraction of inspired oxygen in newborns. Previous studies have reported a gradual decline in neonatal SpO_2_ with increasing altitude [7,8]. For instance, in healthy newborns living at 1800 m, the 5th–95th percentile range of SpO_2_ within 24 hours after birth is 89 %–97 % [9]. Among healthy children, SpO_2_ averages around 90 % at 2500 m and decreases to approximately 85 % at 3200 m [10]. Similar findings were observed in our study. Newborns at high altitudes exhibit relatively lower oxygen saturation, which is a result of multiple coordinated physiological and pathophysiological adaptations rather than a disease state. The underlying mechanisms primarily involve interactions between hypoxic environmental conditions and neonatal physiology: (1) Reduced atmospheric oxygen pressure: As altitude increases, atmospheric pressure and the partial pressure of oxygen (PO_2_) decline proportionally. This reduction decreases alveolar oxygen pressure (PAO_2_) and arterial oxygen pressure (PaO_2_), leading to a corresponding fall in SpO_2_ according to the oxyhemoglobin dissociation curve. (2) Fetal hemoglobin characteristics: Neonates primarily possess fetal hemoglobin, which has a higher oxygen affinity, facilitating oxygen uptake in the lungs but making oxygen release to peripheral tissues more difficult. To compensate, the body allows SpO_2_ to stabilize at a relatively lower yet functionally adequate level, ensuring sufficient oxygen delivery to tissues. Our study found that the rate of SpO_2_ increase within 0–10 minutes, 10–30 minutes, and 60–120 minutes after birth, as well as the final plateau level, varied by altitude. Newborns must rapidly transition from fetal circulation to adult-type circulation immediately after birth to establish normal respiration and systemic-pulmonary blood flow.

At birth, neonates must rapidly transition from the fetal circulation pattern to the normal adult circulation pattern to establish effective respiration and pulmonary blood flow, in order to establish effective respiration and pulmonary blood flow, thereby maintaining normal SpO_2_ levels. A critical step in this transition is the first breath, which expands the alveoli and raises alveolar oxygen partial pressure from the low fetal levels to near-atmospheric levels. The resulting high-oxygen environment inhibits pulmonary vasoconstriction, sharply reducing pulmonary vascular resistance, while systemic vascular resistance rises. As a result, a substantial portion of right heart blood is redirected to the lungs for gas exchange, decreasing right-to-left shunting through the foramen ovale and ductus arteriosus and promoting oxygenated pulmonary blood flow. Concurrently, fetal hemoglobin (HbF) is gradually replaced by adult hemoglobin, which has a lower oxygen affinity, facilitating oxygen release to the tissues and improving oxygen transport efficiency. Increasing blood oxygen levels also induce contraction of the ductus arteriosus smooth muscle, resulting in functional closure within 10–15 hours after birth and anatomical closure within 3–4 days. This effectively eliminates abnormal shunting between the systemic and pulmonary circulations, ensuring adequate delivery of oxygen-rich blood throughout the body. The coordinated operation of these processes allows SpO_2_ levels in healthy neonates to rise steadily during the early postnatal period. At higher altitudes, however, prolonged fetal exposure to hypoxia leads to stronger pulmonary vasoconstriction, slower PVR decline, relatively elevated right heart pressures, and persistent right-to-left shunting. This shunting allows partially deoxygenated blood to enter systemic circulation, “diluting” arterial oxygen content. Consequently, SpO_2_ rises more slowly and plateaus at lower levels than in low-altitude newborns.

The difference in initial oxygen saturation between 552 meters and 1890 meters was small. However, variations in SpO_2_ at different altitudes are the result of multiple interacting factors. During the first two hours after birth, important physiological adjustments occur between the pulmonary and systemic circulations, and as altitude increases, the partial pressure of oxygen in the atmosphere gradually decreases, directly affecting neonatal oxygenation during this critical period. First, the impact of environmental oxygen partial pressure must be considered. At 2416 meters, the partial pressure of oxygen is substantially lower than at 1890 meters, causing PAO_2_ to drop from approximately 65 mmHg to around 55 mmHg. This reduction directly decreases the efficiency of oxygen diffusion in the lungs, resulting in generally lower SpO_2_ in neonates at higher altitudes, with the degree of decline varying according to individual adaptive capacity. Second, ventilation-perfusion mismatch may be exacerbated at high altitudes. Hypoxic pulmonary vasoconstriction can lead to uneven pulmonary perfusion, leaving some alveoli inadequately ventilated. At higher altitudes, such as 2416 meters, this mismatch may be more pronounced, further amplifying the decline in SpO_2_. Third, differences in transitional circulatory adjustments also play a role. After birth, the decrease in pulmonary artery pressure and the closure of the foramen ovale and ductus arteriosus occur dynamically. High-altitude conditions may delay or interfere with these processes. At 2416 meters, severe hypoxic stress may increase the risk of right-to-left shunting, further lowering SpO_2_, whereas at 1890 meters, these adjustments are likely to proceed more smoothly. Finally, HbF, which predominates at this stage, has a high oxygen affinity. However, at high altitude, the lower atmospheric oxygen partial pressure reduces the amount of oxygen available for binding in the lungs, limiting the total oxygen carried by HbF and decreasing SpO_2_. Additionally, neonates have relatively high metabolic rates, increasing their oxygen demand; at high altitudes, restricted oxygen availability further exacerbates the differences in SpO_2_.

Populations residing in high-altitude regions are chronically exposed to hypobaric hypoxia, which has been shown to adversely affect fetal development and neonatal health outcomes [11,12]. Physiological alterations associated with hypoxia typically become apparent at elevations above 1500 meters, with more pronounced effects observed beyond 2500 meters [13]. Epidemiological studies have demonstrated an inverse relationship between altitude and neonatal birth weight, indicating an average reduction of approximately 100 grams for every 1000-meter increase in elevation. The prevalence of low birth weight is significantly higher among neonates born in high-altitude regions compared to those born in lowland areas, implicating altitude as a critical risk factor for low birth weight [14].

Populations with prolonged residence at high altitudes, such as those of Tibetan ethnicity, exhibit adaptive physiological and genetic traits that enhance tolerance to hypoxic environments. Genomic analyses have identified distinct gene-level adaptations in high-altitude populations compared to those without long-term exposure to hypoxia [15]. Comparative data indicate that neonates of Tibetan ethnicity, who are native to high-altitude environments, present with higher birth weights and lower incidence of low birth weight relative to neonates of Han ethnicity who have migrated to high-altitude regions. These findings indicate a population-specific adaptation to hypoxic conditions. Birth weight remains one of the most reliable indicators of population-level environmental adaptation [16,17].

In addition to birth weight, neonatal body length serves as a key anthropometric parameter for assessing physical development at birth. The weight-to-length ratio is considered a more accurate reflection of intrauterine nutritional status and neonatal body composition. In the current dataset, which included healthy full-term neonates born at altitudes ranging from 552 meters to 4,493 meters in the Yushu region, no significant differences were observed in birth weight or weight-to-length ratio across altitudes. However, variations in birth length were identified, potentially attributable to differences in ethnic composition at varying elevations. In areas above 3,000 meters, particularly in Yushu, where the population is predominantly of Tibetan ethnicity, the absence of significant differences in neonatal birth weight and weight-to-length ratio further supports the hypothesis of high-altitude adaptation in this ethnic group.

## Conclusion

SpO_2_ measured within the first two hours post-delivery also varied among full-term neonates born at different altitudes. In neonates born at high altitudes, SpO_2_ values demonstrated a gradual increase during the initial two hours following birth. This postnatal oxygenation pattern, in conjunction with stable anthropometric parameters, underscores the effective physiological adaptation of neonates of Tibetan ethnicity to chronic high-altitude hypoxia.

## Figures and Tables

**Table 1:** General characteristics of neonates by regions

Variables (n=963)	Regions					Test Statistic	*P* value
	552 m	1890 m	2416 m	3280 m	4493 m		
*Sex [n (%)]*						χ^2^=3.82	0.431
*Male*	103 (20.89)	63 (12.78)	195 (39.55)	61 (12.37)	71 (14.40)		
*Female*	112 (23.83)	68 (14.47)	187 (39.79)	46 (9.79)	57 (12.13)		
*Ethnicity [n (%)]*						χ^2^=210.67	<0.001
*Han ethnicity*	94 (27.01)	104 (29.89)	137 (39.37)	13 (3.74)	0 (0.00)		
*Ethnic minorities*	121 (19.67)	27 (4.39)	245 (39.84)	94 (15.28)	128 (20.81)		
*Total [n (%)]*	215 (22.33)	131 (13.60)	382 (39.67)	107 (11.11)	128 (13.29)	-	-

n represents the total number of cases for that variable, and the number in parentheses represents the percentage of that variable relative to the total number of cases.

**Table 2:** Neonatal oxygen saturation (SpO_2_) distribution at different postnatal time points

Variables	Regions					Test Statistic	*P* value
	552 m	1890 m	2416 m	3280 m	4493 m		
*After birth oxygen saturation [M (IQR)]*							
*0* – *10 minutes*	97 (2)	96 (3)	78 (13.75)	84 (20)	73 (4.25)	*H*=694.24	<0.001
*10* – *30 minutes*	97 (3)	98 (2)	89 (5)	90 (2)	79 (7)	*H*=739.76	<0.001
*60* – *120 minutes*	98 (3)	99 (2)	93 (3)	92 (2.5)	95 (3)	*H*=556.76	<0.001
*Total [n (%)]*	215 (22.33)	131 (13.60)	382 (39.67)	107 (11.11)	128 (13.29)	-	-

**Table 3:** Inter-regional differences in neonatal birth anthropometry

Variables	Regions					Test Statistic	*P* value
	552 m	1890 m	2416 m	3280 m	4493 m		
*Birth anthropometry [M (IQR)]*							
*Birth weight (g)*	3300 (500)	3260 (473)	3220 (518)	3280 (425)	3200 (550)	*H*=1.29	0.863
*Body length (cm)*	49 (2)^b^	50 (2)^a^	50 (1)^c^	50 (2)^c^	50 (2)^a^	*H*=38.81	<0.001
*Weight-to-length ratio (kg/m)*	6.67 (0.78)	6.51 (0.69)	6.49 (0.92)	6.60 (0.82)	6.40 (1.10)	*H*=6.05	0.196
*Total [n (%)]*	215 (22.33)	131 (13.60)	382 (39.67)	107 (11.11)	128 (13.29)	-	-
